# Association between prognostic nutritional index and mortality in older Japanese patients with severe post-stroke dysphagia requiring nutritional support: a retrospective cohort study

**DOI:** 10.3389/fneur.2026.1902644

**Published:** 2026-10-19

**Authors:** Wangchun Wu, Jianling Wang

**Affiliations:** Department of Neurosurgery, The Affiliated Mindong Hospital of Fujian Medical University, Fuan, Fujian, China

**Keywords:** C-reactive protein/albumin ratio, mortality, older adults, post-stroke dysphagia, prognostic nutritional index

## Abstract

**Background:**

The prognostic nutritional index (PNI), calculated from serum albumin and lymphocyte count, has remained insufficiently investigated in older patients with post-stroke dysphagia (PSD). We investigated whether PNI was associated with all-cause mortality in older Japanese patients with PSD and compared its prognostic performance with that of the C-reactive protein/albumin ratio (CAR).

**Methods:**

This single-center retrospective cohort study included 132 older Japanese patients with severe PSD requiring PEG or TPN between January 2014 and January 2017. Multivariable Cox regression analysis and Kaplan–Meier curves were used to estimate the associations of PNI and CAR with all-cause mortality. We assessed robustness through subgroup and sensitivity analyses. To evaluate the prognostic discrimination for 1-year mortality, we used time-dependent receiver operating characteristic (ROC) curves and assessed incremental predictive value using integrated discrimination improvement (IDI) and net reclassification improvement (NRI).

**Results:**

The mean age of the cohort was 80.7 years (78 women, 54 men). In fully adjusted models, multivariable Cox regression showed that each 1-point increment in PNI was associated with a 7% reduction in all-cause mortality (HR = 0.93, 95% CI: 0.90–0.97). Compared with the lowest PNI tertile (T1), the highest tertile (T3) showed a 75% risk reduction (HR = 0.25; 95% CI: 0.10–0.60). For 1-year mortality, the time-dependent AUC was numerically higher for PNI than for CAR (0.771 vs. 0.712; *p* = 0.16). Adding PNI to a clinical baseline model significantly improved discrimination (IDI = 0.055, 95% CI 0.010–0.104, *p* < 0.001; NRI = 0.471, 95% CI 0.126–0.957), whereas adding CAR did not (IDI = 0.015, *p* = 0.31).

**Conclusion:**

PNI was inversely associated with mortality in older Japanese patients with severe post-stroke dysphagia requiring PEG or TPN and provided incremental prognostic information beyond standard clinical variables. Whether PNI may be useful for risk stratification in this population requires prospective and external validation.

## 1 Introduction

Post-stroke dysphagia (PSD) is defined as swallowing dysfunction resulting from damage to the neural swallowing network following stroke (1). It is a common but frequently under-recognized complication of stroke (2). A 2024 meta-analysis reported an overall PSD prevalence of 46.6%, with a prevalence of approximately 40.1% in Asian populations (3). PSD increases the risk of aspiration pneumonia, malnutrition, and prolonged hospitalization, thereby contributing to poor prognosis and higher mortality (4, 5). With rapid global population ageing, PSD is particularly prevalent among older adults (6), making the identification of reliable prognostic markers critical for clinical practice.

Composite biomarkers have garnered increasing attention as practical tools for prognostic stratification. The C-reactive protein (CRP)-to-albumin ratio (CAR), which integrates systemic inflammation (as reflected by CRP) and nutritional reserve (indicated by albumin), has demonstrated independent prognostic value for short- and long-term mortality across a broad spectrum of diseases, including cancer (7), critical illness (8), and stroke (9). A recent study of older patients with post-stroke dysphagia demonstrated that elevated CAR levels were independently associated with increased all-cause mortality (10). The Prognostic Nutritional Index (PNI), another widely used metric for prognostic prediction across multiple diseases (11), is calculated from serum albumin and the total lymphocyte count to reflect a patient’s nutritional and immune status. Previous studies have demonstrated that a low PNI is associated with increased mortality and poorer prognosis in several diseases (12), including increased in-hospital mortality and worse 3-month functional outcomes in patients with acute ischemic stroke (13, 14). Among these, PNI has also been examined in older patients with dysphagia, but in a mixed-etiology cohort derived from the same dataset as the present study. All prior analyses of this dataset are summarized in Supplementary Table S1. However, whether PNI has prognostic value specifically in older PSD populations and whether it offers better discriminatory power than CAR remain unclear. To date, no study has directly compared the predictive performance of PNI and CAR within a single cohort.

To address this gap, the primary aim of this study was to evaluate the association between PNI and all-cause mortality in a cohort of older Japanese patients with PSD. The secondary aim was to compare the predictive accuracy of PNI and CAR for 1-year mortality. Given its incorporation of both nutritional and immunological components, we hypothesized that a lower PNI would be associated with a higher mortality risk and that PNI would demonstrate better prognostic discrimination than CAR.

## 2 Materials and methods

### 2.1 Data source

This study was a secondary analysis of publicly accessible data. The dataset was retrieved from the Dryad Digital Repository, originally published by Masaki and Kawamoto (15). This study was reviewed and approved by the Ethics Review Committee. Given that all data were fully anonymized before release, informed consent was waived. We obtained the study data from the Dryad Digital Repository: https://datadryad.org/dataset/doi:10.5061/dryad.gg407h1.

### 2.2 Study design and population

This single-center retrospective cohort study included older adults with severe dysphagia who received either percutaneous endoscopic gastrostomy (PEG) or total parenteral nutrition (TPN) at Miyanomori Memorial Hospital (Sapporo, Japan) between January 2014 and January 2017. Dysphagia was diagnosed by a multidisciplinary team-including physicians, nurses, and speech-language pathologists-based on clinical assessment and videofluoroscopic swallowing studies. Patients were excluded if they had advanced malignancy, required PEG for gastric decompression, or had undergone PEG placement before January 2014. The initial cohort comprised 253 patients. To address the study objective, only individuals with a documented history of stroke were retained for analysis, resulting in a final cohort of 133 patients with post-stroke dysphagia. One was excluded due to missing lymphocyte count (required for PNI calculation), yielding a final cohort of 132 patients with complete data for all variables (Figure 1). Among the 132 patients included in the analysis, no variable had missing values; the analysis is therefore a complete-case analysis with no imputation. In this study, post-stroke dysphagia was defined operationally as severe dysphagia requiring PEG or TPN in patients with a documented history of stroke, consistent with prior analyses of this dataset (10). Stroke is the principal recorded neurological cause of dysphagia in this cohort; however, because the dataset records cerebrovascular disease only as a binary variable and does not include stroke timing, subtype, severity, or lesion location, a causal attribution of dysphagia to stroke could not be established.

**Figure 1:**
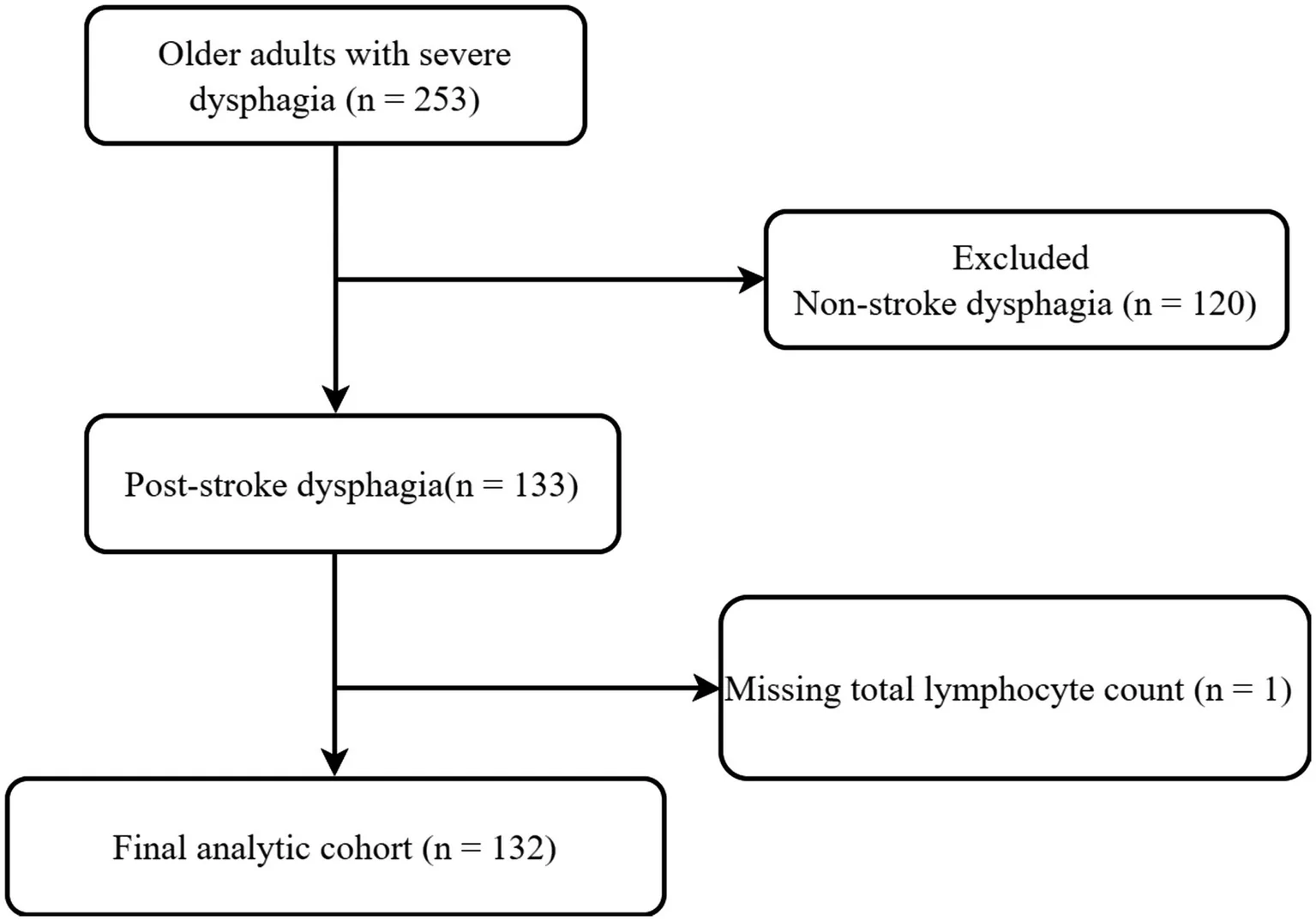
Patients’ flow chart.

### 2.3 Variable definitions

The primary endpoint was all-cause mortality. Follow-up began on the date of nutritional support initiation and continued until death or the conclusion of the study (28 February 2018). Covariates included demographic factors (age, sex), modality of nutritional support (PEG vs. TPN), comorbidities (severe dementia, aspiration pneumonia, ischemic heart disease [IHD]), and Clinical Frailty Scale (CFS) score (dichotomized as ≥8 versus <8 because only one patient had a score of 4.). Because recovery of oral intake occurs after baseline and could introduce immortal-time bias, it was not included in the adjusted models. Multicollinearity was assessed using the generalized variance inflation factor (GVIF), adjusted for degrees of freedom (DF). A GVIF^(1/(2 × DF)) ≥ 2 was considered indicative of significant collinearity. All adjusted GVIF values were below this threshold, indicating no multicollinearity among study variables (Supplementary Table S2). Blood samples were collected within 7 days before initiating PEG feeding or TPN. The following indices were categorized into tertiles based on their distributions in the cohort: PNI: T1 < 36.7; T2 36.7–42.8; T3 ≥ 42.8. C-reactive protein/albumin ratio (CAR): T1 < 0.092; T2 0.092–0.592; T3 ≥ 0.592. The formulas applied were the following (16):

$$\begin{array}{l} \begin{array}{l} \mathrm{PNI}=\text{serum albumin}\;(g/\mathrm{dL})\times 10+\text{total}\;\\ \text{lymphocyte count}\;(/{\mathrm{mm}}^{3})\times 0.005; \end{array}\\ \mathrm{CAR}=C-\text{reactive protein}\;(\mathrm{mg}/\mathrm{dL})/\text{serum albumin}\;(g/\mathrm{dL}) \end{array}$$

### 2.4 Statistical analysis

Continuous variables were reported as mean (standard deviation [SD]) or median [interquartile range (IQR)], depending on their distribution. Categorical variables were reported as frequencies and corresponding percentages (*n*, %). Between-group comparisons were performed using one-way analysis of variance (ANOVA) for normally distributed variables and the Kruskal–Wallis test for skewed variables, and the chi-square test or Fisher’s exact test for categorical variables, as appropriate. Cox proportional hazards regression was used to examine the relationships of PNI and CAR with mortality. Hazard ratios (HRs) and 95% confidence intervals (CIs) were calculated. Three sequentially adjusted models were fitted: Model 1, unadjusted; Model 2, adjusted for age and sex; and Model 3, further adjusted for age, sex, PEG, severe dementia, aspiration pneumonia, ischemic heart disease, CFS score. The proportional hazards assumption was evaluated using Schoenfeld residuals tests and the corresponding global goodness-of-fit test; the test statistics are reported in Supplementary Table S3 and Supplementary Figure S1, and the assumption was met in the fully adjusted models (all *p* > 0.05). Survival probabilities were estimated using the Kaplan–Meier method, and group differences were assessed with the log-rank test. As a supplementary analysis that does not rely on the proportional hazards assumption, restricted mean survival time (RMST) was compared across PNI tertiles at fixed time points. Median follow-up was estimated using the reverse Kaplan–Meier method. Because some patients were censored before 1 year, the discriminatory capacity for 1-year mortality was assessed with time-dependent ROC analysis (Uno’s method), and the IDI and NRI were computed using censoring-aware methods (survIDINRI and nricens). To assess incremental prognostic value, we compared a clinical baseline model (age, sex, PEG, severe dementia, aspiration pneumonia, ischemic heart disease, CFS) with the same model after adding PNI, after adding CAR, and after adding both, using likelihood-ratio tests, AIC, C-index, Brier score, and time-dependent ROC. The incremental prognostic value was further quantified using the Integrated Discrimination Improvement (IDI) and the Net Reclassification Improvement (NRI). Decision curve analysis (DCA) was used to assess clinical net benefit across a range of threshold probabilities. Subgroup analyses were performed, stratified by age, sex, PEG status, severe dementia, aspiration pneumonia, and ischemic heart disease. Interaction effects were evaluated using the likelihood ratio test. Two sensitivity analyses were conducted. Restricted cubic spline models were used to test for potential non-linear associations of PNI and CAR with mortality. Model calibration was assessed with a bootstrap calibration curve, and overall prediction error was quantified with the Brier score. Internal validation was performed by bootstrap resampling (500 resamples) to estimate the optimism-corrected C-index. Given the limited number of events (61), a Firth penalized Cox model was also fitted to reduce small-sample bias. All statistical analyses were performed using R^1^ and Free Statistics (version 2.4). All tests were two-tailed, and *p* < 0.05 was considered statistically significant.

## 3 Results

### 3.1 Baseline characteristics of the study population

In this cohort of 132 patients with post-stroke dysphagia (mean age, 80.7 ± 10.3 years; 59.1% female), 107 (81.1%) received PEG and 25 (18.9%) received TPN. The proportion receiving PEG increased across PNI tertiles (T1: 65.9%, T2: 81.8%, T3: 95.5%; *p* = 0.002). Significant differences across tertiles were observed for age, severe dementia, CFS score, and mortality (all *p* < 0.05). Notably, mortality during follow-up declined progressively with higher PNI tertiles (T1 65.9%, T2 54.5%, T3 18.2%; *p* < 0.001; Table 1).

**Table 1:** Baseline characteristics of patients.

Variables	Total (*n* = 132)	PNI			*p*
		T1 (<36.7)	T2 (36.7–42.8)	T3 (≥42.8)	
Age(year), Mean ± SD	80.7 ± 10.3	84.6 ± 8.2	81.8 ± 8.2	75.5 ± 12.2	< 0.001
Sex, *n* (%)					0.168
Male	54 (40.9)	20 (45.5)	21 (47.7)	13 (29.5)	
Female	78 (59.1)	24 (54.5)	23 (52.3)	31 (70.5)	
PEG, *n* (%)	107 (81.1)	29 (65.9)	36 (81.8)	42 (95.5)	0.002
Severe dementia, *n* (%)	17 (12.9)	10 (22.7)	5 (11.4)	2 (4.5)	0.037
Aspiration pneumonia, *n* (%)	32 (24.2)	14 (31.8)	12 (27.3)	6 (13.6)	0.117
Ischemic heart diseases, *n* (%)	29 (22.0)	14 (31.8)	9 (20.5)	6 (13.6)	0.115
CFS score, *n* (%)					0.008
< 8	18 (13.6)	6 (13.6)	1 (2.3)	11 (25)	
≥ 8	114 (86.4)	38 (86.4)	43 (97.7)	33 (75)	
Oral intake recovery, *n* (%)	8 (6.1)	1 (2.3)	1 (2.3)	6 (13.6)	0.051
CRP (mg/dL), Median (IQR)	0.8 (0.2, 2.4)	2.2 (1.1, 5.4)	0.9 (0.3, 2.3)	0.2 (0.1, 0.4)	< 0.001
Alb(g/dL), Mean ± SD	3.3 ± 0.6	2.6 ± 0.4	3.4 ± 0.2	3.8 ± 0.4	< 0.001
TLC (cells /mm^3^), Mean ± SD	1416.4 ± 727.7	993.2 ± 381.0	1314.0 ± 438.3	1941.9 ± 894.2	< 0.001
PNI, Mean ± SD	39.7 ± 7.8	31.0 ± 4.6	40.1 ± 1.6	47.9 ± 3.8	< 0.001
CAR, Median (IQR)	0.2 (0.1, 0.9)	1.0 (0.4, 2.0)	0.3 (0.1, 0.7)	0.1 (0.0, 0.1)	< 0.001
Status, *n* (%)					< 0.001
Alive	71 (53.8)	15 (34.1)	20 (45.5)	36 (81.8)	
Dead	61 (46.2)	29 (65.9)	24 (54.5)	8 (18.2)	

PEG, percutaneous endoscopic gastrostomy; CFS, Clinical Frailty Scale scores; CRP, C-reactive protein; Alb, albumin; TLC, total lymphocyte count; PNI, prognostic nutritional index; CAR: C-reactive protein/albumin ratio; SD: standard deviation; IQR: interquartile range.

### 3.2 Survival analysis

Restricted cubic spline analyses showed no evidence of a non-linear association for either index (Supplementary Figure S2). Median follow-up (reverse Kaplan–Meier) was 714 days; 69 of 132 patients (52.3%) had at least 1 year of follow-up, 44 died within 1 year, and 19 were censored alive before 1 year. The Kaplan–Meier curves showed statistically significant differences in survival across tertiles of PNI and CAR (all log-rank *p*-values < 0.0001). For PNI, the median survival duration was as follows: 247 days for T1, 699 days for T2, and had not been reached for T3 by the end of the maximum follow-up period (1,463 days). For CAR, the median survival was 282 days at T3, 739 days at T2, and had not yet been reached at T1 (Figure 2). Brief crossing of the T2 and T3 curves near the end of follow-up reflected the very small number of patients still at risk (3 to 4 per group) rather than a violation of the proportional hazards assumption, which was met in the fully adjusted models (all Schoenfeld *p* > 0.05; Supplementary Table S3). In an assumption-free restricted mean survival time analysis, survival time increased monotonically across PNI tertiles at 1 year (T1: 225 days; T2: 289 days; T3: 359 days; T3 vs. T1 difference: 134 days, 95% CI 88–178) and at 2 years (difference: 333 days, 95% CI 236–424). Cox proportional hazards regression analyses were performed to evaluate the associations of PNI and CAR with mortality (Table 2). In the unadjusted model (Model 1), PNI analyzed as a continuous variable was significantly inversely associated with mortality: every 1-point increment in PNI was associated with a 9% lower risk of death (HR = 0.91, 95% CI: 0.88–0.94; *p* < 0.001). In the fully adjusted model (Model 3), the HR was 0.93 (95% CI: 0.90–0.97; *p* < 0.001). In categorical analysis of PNI, after adjusting for all covariates, the T3 (highest) group had a 75% lower mortality risk than the T1 (lowest) group (HR = 0.25; 95% CI: 0.10–0.60; *p* = 0.002). In contrast, CAR as a continuous variable was positively associated with mortality (HR = 1.38, 95% CI: 1.07–1.78, *p* = 0.012). Patients in the highest tertile (T3) had a 3.10-fold higher mortality risk than those in the lowest tertile (T1) (95% CI: 1.36–7.02, *p* = 0.007). In a Firth penalized Cox model, PNI remained significantly inversely associated with mortality (HR 0.93, 95% CI 0.90–0.97, *p* < 0.001). Bootstrap internal validation showed minimal optimism (apparent C-index 0.821; optimism-corrected 0.810; Supplementary Table S4).

**Figure 2:**
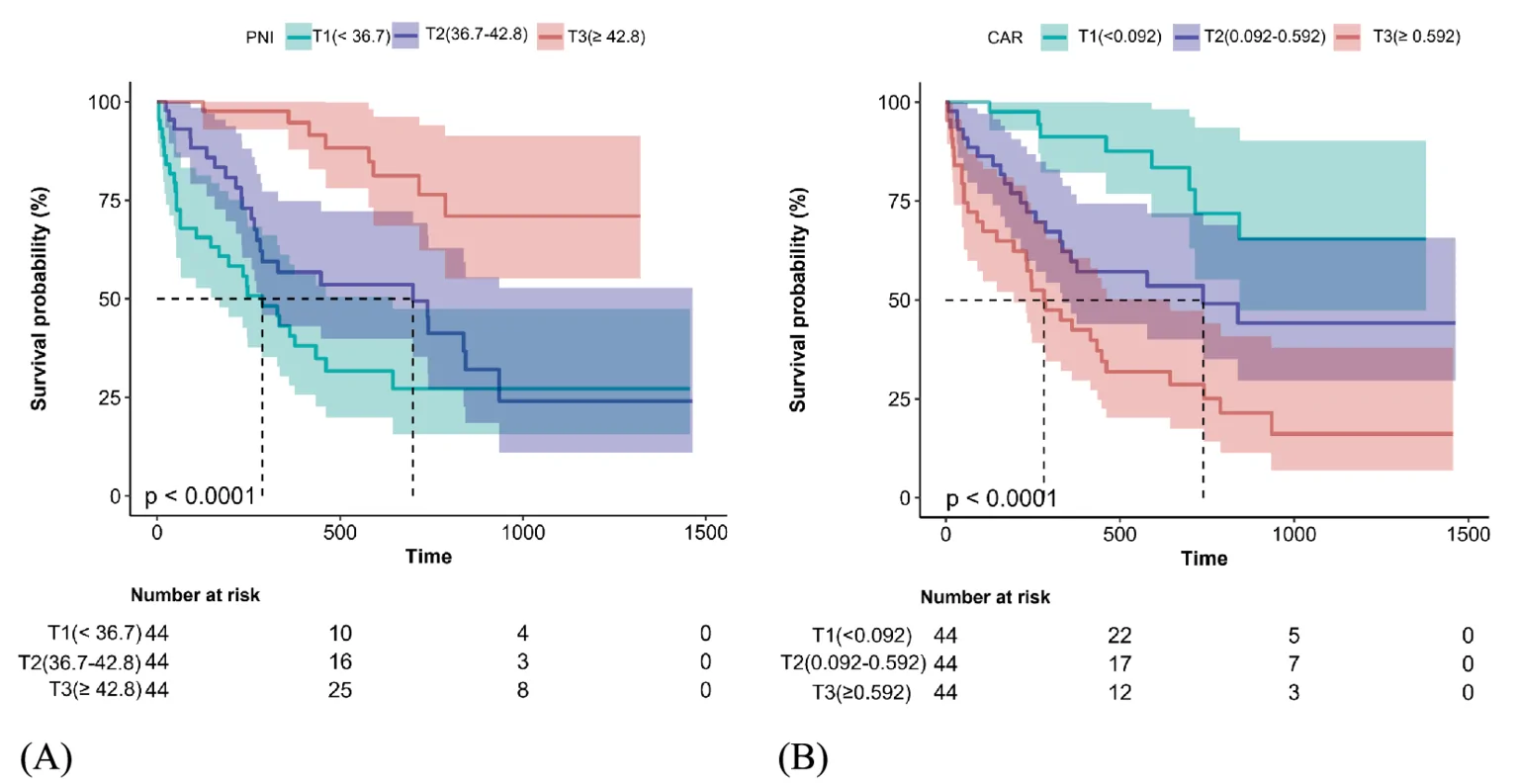
Kaplan–Meier survival analysis for all-cause mortality stratified by the PNI **(A)** and the CAR **(B)** in older patients with post-stroke dysphagia. Shaded areas represent 95% confidence intervals. PNI, Prognostic Nutritional Index. CAR, C-reactive protein-to-albumin ratio.

**Table 2:** Association between PNI, CAR and mortality in different models.

Variable	Model 1		Model 2		Model 3	
	HR (95%CI)	*p*-value	HR (95%CI)	*p*-value	HR (95%CI)	*p*-value
PNI (continuous)	0.91 (0.88–0.94)	<0.001	0.92 (0.89–0.95)	<0.001	0.93 (0.90–0.97)	<0.001
PNI (T1, < 36.7)	1(Ref)		1(Ref)		1(Ref)	
PNI (T2, 36.7–42.8)	0.61 (0.36 ~ 1.06)	0.077	0.6 (0.34 ~ 1.06)	0.077	0.46 (0.25 ~ 0.83)	0.01
PNI (T3, ≥ 42.8)	0.16 (0.07 ~ 0.34)	<0.001	0.25 (0.11 ~ 0.57)	0.001	0.25 (0.1 ~ 0.6)	0.002
*p* for trend	0.44 (0.31 ~ 0.61)	<0.001	0.52 (0.36 ~ 0.76)	0.001	0.49 (0.32 ~ 0.74)	0.001
CAR (continuous)	1.76 (1.39 ~ 2.23)	<0.001	1.57 (1.23 ~ 2.00)	<0.001	1.38 (1.07 ~ 1.78)	0.012
CAR (T1, < 0.092)	1(Ref)		1(Ref)		1(Ref)	
CAR (T2, 0.092–0.592)	2.91 (1.29 ~ 6.57)	0.01	2.21 (0.96 ~ 5.08)	0.062	3.03 (1.25 ~ 7.34)	0.014
CAR (T3, ≥0.592)	5.8 (2.67 ~ 12.6)	<0.001	4.02 (1.83 ~ 8.87)	0.001	3.18 (1.40 ~ 7.23)	0.006
*p* for trend	2.3 (1.63 ~ 3.24)	<0.001	1.96 (1.37 ~ 2.79)	<0.001	1.59 (1.12 ~ 2.26)	0.009

Model 1: Non-adjusted. Model 2: Adjusted for age, sex. Model 3: Adjusted for age, sex, percutaneous endoscopic gastrostomy, severe dementia, aspiration pneumonia, ischemic heart disease, clinical frailty scale scores (binary). PNI: prognostic nutritional index; CAR: C-reactive protein/albumin ratio; CI, confidence interval; HR: hazard ratio.

### 3.3 Comparison of predictive performance

For 1-year mortality, as a single marker, the time-dependent AUC was numerically higher for PNI (0.771, 95% CI 0.686–0.856) than for CAR (0.712, 95% CI 0.617–0.808), although the difference was not statistically significant (*p* = 0.16; Figure 3). To assess incremental prognostic value beyond standard clinical variables, we compared a clinical baseline model with the same model after adding PNI or CAR (Table 3). Adding PNI significantly improved model fit (likelihood-ratio *χ*^2^ = 13.30, *p* < 0.001), whereas adding CAR produced a smaller improvement (*χ*^2^ = 5.55, *p* = 0.018). PNI added significant information on top of the CAR-containing model (*χ*^2^ = 8.00, *p* = 0.005), whereas CAR did not add information on top of PNI (*χ*^2^ = 0.25, *p* = 0.62). Discrimination (C-index 0.821 vs. 0.800) and overall prediction error (Brier score 0.145 vs. 0.160) both favored the PNI model. In censoring-aware analyses, PNI significantly improved discrimination and reclassification over the clinical model (IDI = 0.055, 95% CI 0.010–0.104, *p* < 0.001; NRI = 0.471, 95% CI 0.126–0.957), whereas CAR did not (IDI = 0.015, *p* = 0.31). Model calibration was acceptable, with observed 1-year mortality tracking predicted risk closely across quartiles of predicted risk (observed 0.0%, 18.5%, 44.0%, and 79.4% vs. predicted 5.5%, 14.9%, 36.5%, and 81.8%, respectively; Supplementary Figure S3). Decision-curve analysis suggested that both PNI and CAR provided a positive net benefit versus the “treat-all” and “treat-none” strategies across a range of threshold probabilities (Supplementary Figure S4), although this should be interpreted cautiously in the absence of external validation.

**Figure 3:**
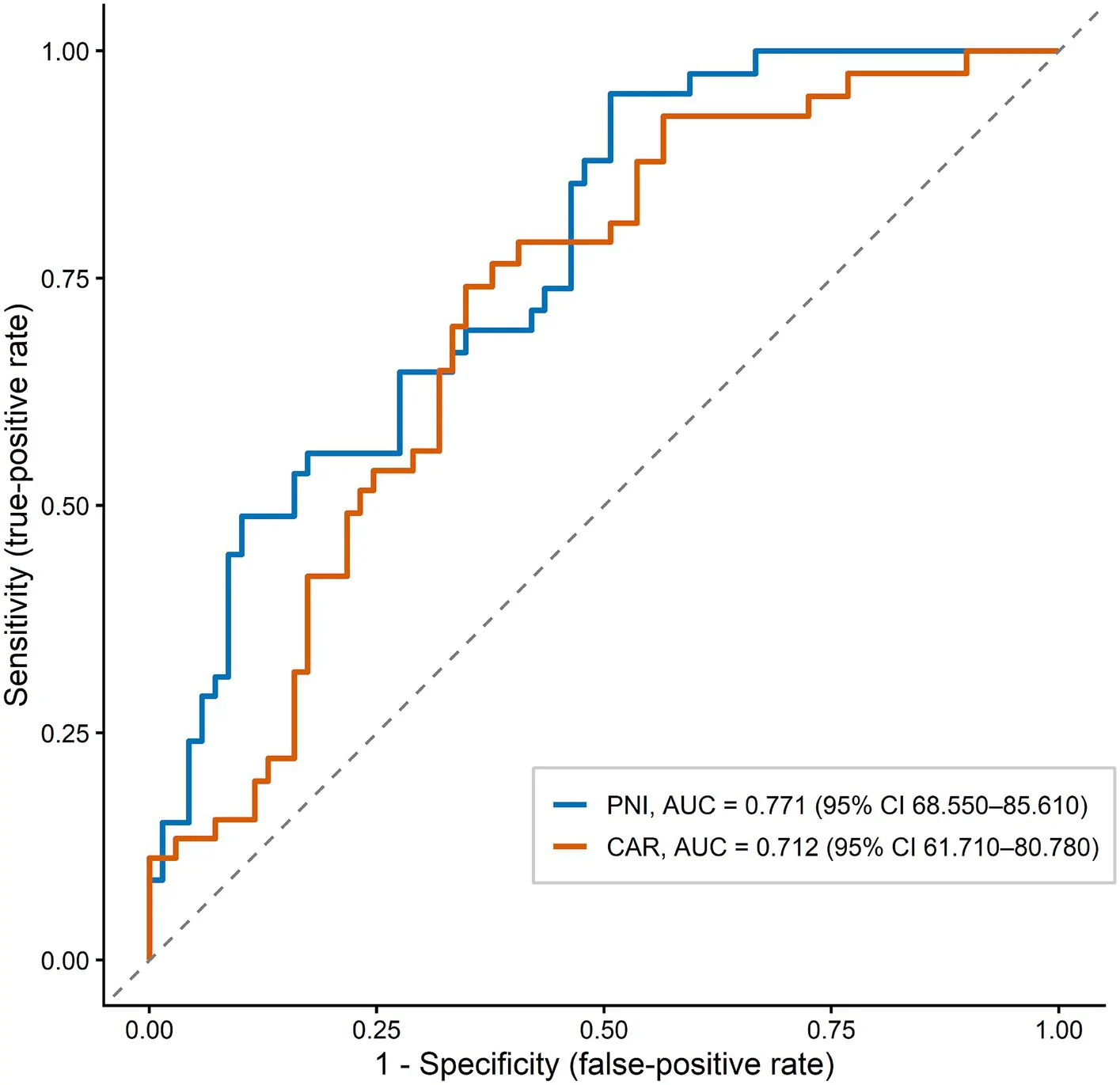
Time-dependent receiver operating characteristic (ROC) curves for the prediction of 1-year mortality (Uno’s estimator). AUC, area under the curve. PNI, Prognostic Nutritional Index. CAR, C-reactive protein-to-albumin ratio.

**Table 3:** Incremental prognostic value of PNI and CAR for 1-year mortality beyond a clinical baseline model.

Metric	Clinical model	Clinical + PNI	Clinical + CAR	Clinical + PNI + CAR
C-index (concordance)	0.800	0.821	0.806	0.821
Time-dependent AUC (1 year)	0.836	0.865	0.842	0.862
Brier score (1 year)	0.160	0.145	0.158	0.146
AIC	469.3	458.0	465.7	459.7
LR test vs. clinical model	—	*χ*^2^ = 13.30, *p* < 0.001	*χ*^2^ = 5.55, *p* = 0.019	*χ*^2^ = 13.55, *p* = 0.001
IDI vs. clinical model (95% CI, *p*)	Reference	0.055 (0.010–0.104), *p* < 0.001	0.015 (−0.008–0.063), *p* = 0.31	—
Continuous NRI vs. clinical model (95% CI)	Reference	0.471 (0.126–0.957)	0.477 (−0.125–0.767)	—

The clinical model included age, sex, PEG, severe dementia, aspiration pneumonia, ischemic heart disease, and CFS (binary). PNI added significant information on top of the CAR-containing model (*χ*^2^ = 8.00, *p* = 0.005), whereas CAR did not add information on top of PNI (*χ*^2^ = 0.25, *p* = 0.62). PNI, prognostic nutritional index; CAR, C-reactive protein/albumin ratio; AUC, area under the receiver operating characteristic curve; LR, likelihood ratio; IDI, integrated discrimination improvement; NRI, net reclassification improvement; CI, confidence interval.

### 3.4 Subgroup analyses and sensitivity analyses

Subgroup analyses were performed to assess the consistency of the association between PNI and mortality across different patient subgroups (Figure 4). The inverse association between PNI and mortality remained consistent across subgroups defined by age (< 85 vs. ≥ 85 years), sex, PEG status, severe dementia, aspiration pneumonia, and ischemic heart disease. No significant interactions were observed (all *p* for interaction > 0.05), suggesting the association was stable across these clinically relevant subgroups. We conducted two sensitivity analyses: one using an alternative PNI cut-off (17), and the other excluding patients who died within the first 30 days of follow-up. Both analyses produced results consistent with the primary analysis (Supplementary Tables S5, S6).

**Figure 4:**
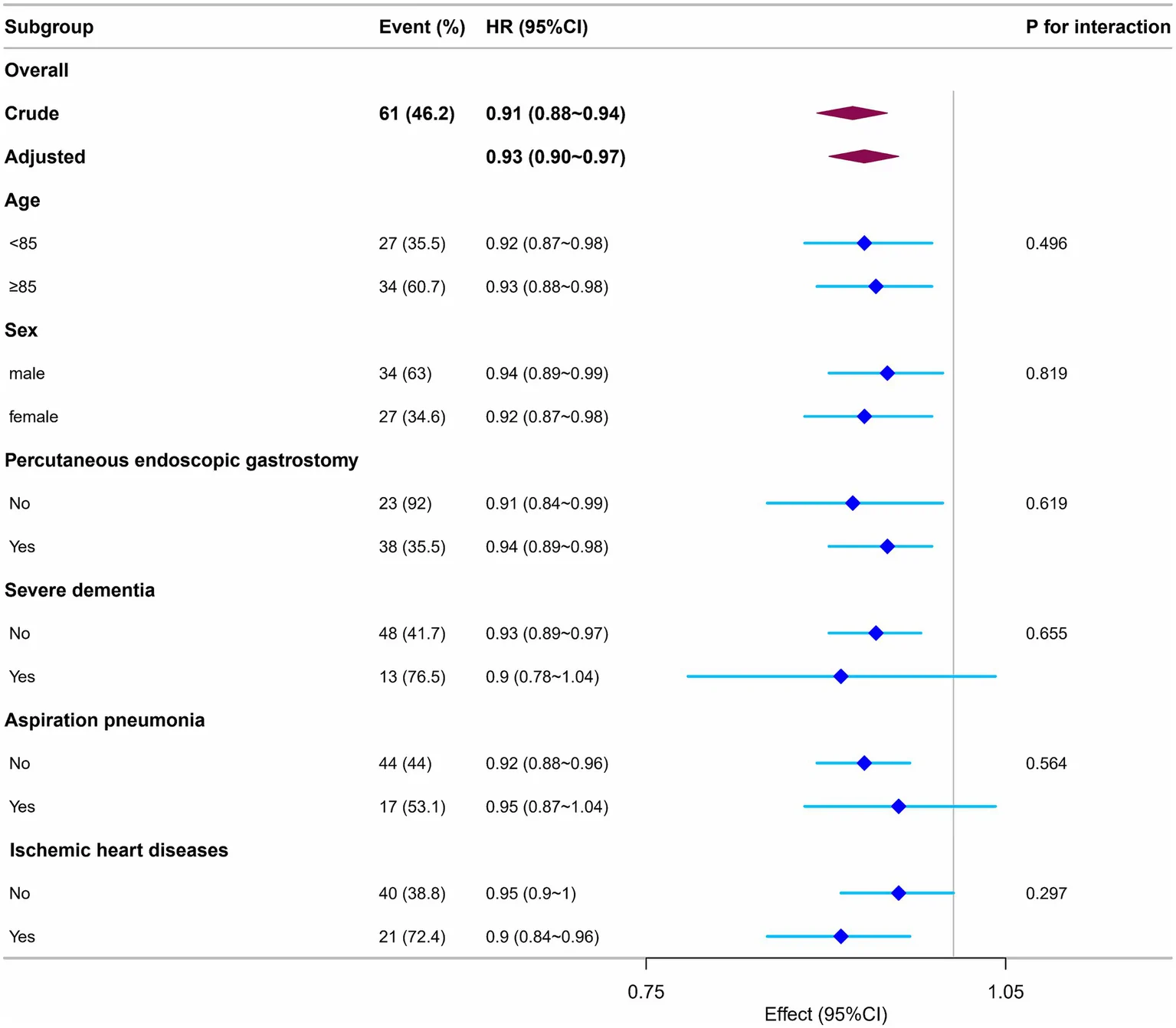
Subgroup analyses for the association between PNI and all-cause mortality. HR, hazard ratio; CI, confidence interval; PNI, Prognostic Nutritional Index.

## 4 Discussion

Swallowing is a complex neuromuscular coordination process governed by multiple regions of the central nervous system. Post-stroke dysphagia results from stroke-induced damage to the neural pathways governing swallowing, impairing the oral, pharyngeal, or esophageal phases of swallowing and attenuating airway protection mechanisms such as the cough reflex (3, 18). Although this mechanistic account describes the typical pathophysiology of post-stroke dysphagia, in the present cohort stroke is treated as the principal recorded neurological cause and we do not claim it was the sole cause in every case, as coexisting contributors such as dementia, neurodegeneration, or deconditioning could not be formally excluded. The incidence detected by instrumental assessment can be as high as 64–78%; it is higher following hemorrhagic stroke (58.8%) than after ischemic stroke (43.6%) and shows geographic variation (3, 19). Studies report that 43–54% of patients with PSD develop aspiration, approximately 37% of whom develop pneumonia; the risk of aspiration pneumonia is 11.5-fold higher than in the general population (20). In a separate literature review, the prevalence of malnutrition among patients with post-stroke dysphagia was approximately 8–34% (21). Such complications impair patients’ quality of life and are associated with markedly increased mortality.

In this analysis of a retrospective cohort of older Japanese patients with post-stroke dysphagia (PSD), we found that a lower Prognostic Nutritional Index was independently associated with higher all-cause mortality after adjustment for the demographic and clinical variables available in the dataset. Specifically, every 1-point increment in PNI was associated with a 7% lower risk of death in the fully adjusted model (HR = 0.93, 95% CI: 0.90–0.97; *p* < 0.001). When analyzed categorically, patients in the highest tertile exhibited a 75% lower mortality risk compared with the lowest tertile (HR = 0.25, 95% CI: 0.10–0.60; *p* = 0.002). This pronounced risk gradient highlights the clinical value of PNI-based stratification and points to its potential for identifying high-risk patients who may derive benefit from more intensive nutritional and supportive interventions.

Aspiration is a major risk factor for aspiration pneumonia, while feeding difficulties directly cause nutritional deficiencies (22, 23). Stroke itself induces systemic inflammation and immunosuppression (24), which may be further exacerbated by aspiration pneumonia. Persistent inflammation and immunosuppression may increase energy and protein requirements, promote catabolism, and accelerate nutrient depletion (25). Malnutrition may in turn weaken immune function, delay neural recovery, and hinder swallowing rehabilitation, potentially forming a vicious cycle that contributes to poor prognosis (26). In this pathophysiological context, PNI, based on serum albumin and total lymphocyte count, may serve as a sensitive indicator of the combined burden of post-stroke malnutrition and immune dysfunction. Albumin and lymphocyte counts are, however, influenced by factors beyond nutritional status (inflammation, infection, hydration, chronic illness, acute stress), so PNI should be viewed as an integrative marker; the underlying mechanisms remain speculative and the retrospective design does not permit causal conclusions.

Furthermore, by directly comparing PNI and CAR in the same cohort, our study further demonstrated that PNI exhibited more favorable prognostic discrimination compared with CAR. The time-dependent AUC was numerically higher for PNI than for CAR (0.771 vs. 0.712, *p* = 0.16). More importantly, PNI significantly improved prediction beyond the clinical baseline model, whereas CAR did not. CAR reflects the interplay between systemic inflammation and nutritional status and is an established determinant of post-stroke outcomes (27). Wang et al. demonstrated that CAR was independently associated with mortality in older adults with PSD, underscoring the prognostic relevance of the inflammation–nutrition axis in this population (10). However, CAR does not capture the immunological dimension. Stroke is thought to induce immunosuppression, predominantly through a shift in lymphocyte phenotype from Th1 to Th2 and by reducing lymphocyte and NK(natural killer) cell counts in peripheral blood and spleen (28). Peripheral lymphocyte counts, which reflect the severity of stroke-induced immunodepression (22), may offer prognostic information beyond that provided by CAR alone. The incremental value of PNI over CAR may partly arise from its incorporation of lymphocyte count, which captures stroke-induced immunosuppression beyond the inflammation-nutrition axis reflected by albumin alone. Our findings indicate that the nutritional-immune profile captured by PNI maintained stable prognostic relevance throughout follow-up, whereas the prognostic value of the inflammatory-nutritional axis, as reflected by CAR, appeared attenuated over time. Because the timing of stroke relative to blood sampling is unknown in this dataset, this temporal pattern is speculative; it may reflect a transition from an acute-phase inflammatory response (dominated by CRP release) toward chronic immune dysregulation and nutritional depletion (captured by lymphopenia and hypoalbuminemia), a hypothesis that requires confirmation in studies with known stroke timing. Whether immunocompetence, as reflected by lymphocyte counts, becomes increasingly important for long-term outcomes in PSD likewise merits prospective evaluation. Collectively, these results indicate that PNI may be a useful prognostic marker in this population, although its clinical value requires evaluation in prospective studies.

The PNI quantifies nutritional and immune status (16) and serves as a practical tool for perioperative risk management, prognostic stratification in cancer and outcome prediction in selected non-neoplastic conditions (29, 30). Previous studies have reported that a low PNI is associated with increased in-hospital mortality and worse 3-month functional outcomes in patients with acute ischemic stroke (13, 14). These consistent findings support PNI as a robust prognostic marker in cerebrovascular disease. Wang et al. reported an inverse association between PNI and mortality in a general dysphagia cohort; however, their analysis included patients with dysphagia of all etiologies (31). We specifically studied older patients with severe dysphagia and a documented history of stroke, in whom the interplay between neurogenic dysphagia, aspiration risk, and immune dysfunction is particularly pronounced. To compare their prognostic discrimination, we directly evaluated PNI and CAR within the same PSD cohort. To our knowledge, this is the first study specifically demonstrating the prognostic utility of PNI in a cohort restricted to patients with PSD.

PSD patients frequently present with multiple comorbidities that complicate risk stratification. We therefore conducted subgroup analyses stratified by age, sex, PEG status, severe dementia, aspiration pneumonia and ischemic heart disease. The inverse association between PNI and mortality persisted across all strata, with no significant interactions observed. In sensitivity analyses using alternative PNI cut-offs and excluding early deaths to minimize bias from early mortality, the prognostic value of PNI remained robust. Decision-curve analysis further indicated that PNI-based risk prediction confers a net clinical benefit across a range of relevant threshold probabilities, although this requires cautious interpretation in the absence of external validation. Whether patients with a PNI in the lowest tertile would benefit from intensified nutritional support guided by this threshold cannot be determined from the present analysis and requires evaluation in prospective interventional trials. PNI offers the practical advantages of simplicity and feasibility: serum albumin and total lymphocyte count are commonly used blood tests in clinical practice that do not require additional specialized testing or impose a financial burden.

There are several limitations to consider. First, the cohort was highly selected, comprising older Japanese patients with severe dysphagia requiring PEG or TPN (81% PEG; 86% with CFS ≥ 8) from a single center. The findings should therefore not be generalized to all stroke patients, those with milder dysphagia, or patients who can continue oral feeding; validation in prospective multicenter cohorts is required. Second, PNI was measured at a single time point, and we cannot assess the prognostic significance of longitudinal PNI trajectories. Third, important clinical variables were unavailable, including stroke subtype, severity, timing, and lesion location, dysphagia severity, pre-stroke functional status, and detailed information on infection and comorbidities. Consequently, dysphagia could not be confirmed as stroke-caused, competing etiologies could not be excluded, and residual confounding from these unmeasured factors cannot be ruled out despite adjustment for the available covariates. Fourth, although we simplified the model, applied penalized regression, and confirmed minimal optimism by bootstrap validation, the number of events (61) remains limited relative to the number of covariates, so the estimates should be interpreted cautiously and require validation in larger external cohorts.

## 5 Conclusion

In this retrospective cohort of older Japanese patients with severe post-stroke dysphagia requiring PEG or TPN, PNI was inversely associated with all-cause mortality and provided incremental prognostic information beyond standard clinical variables, whereas CAR did not. Whether PNI may be useful for risk stratification in this population requires prospective and external validation.

## Data Availability

The datasets presented in this study can be found in online repositories. The names of the repository/repositories and accession number(s) can be found at: https://datadryad.org/dataset/doi:10.5061/dryad.gg407h1.

## Footnotes

1 version 4.2.2; https://www.r-project.org.
