# Supplementary material for: Association between prognostic nutritional index and mortality in older Japanese patients with severe post-stroke dysphagia requiring nutritional support: a retrospective cohort study
Source: Front Neurol. 2026 Oct 19;17:1902644. doi: 10.3389/fneur.2026.1902644 (PMC13724426; doi:10.3389/fneur.2026.1902644)
Supplement: Supplementary file 1 [file Supplementary_file_1.DOCX]

**Supplementary Table S1. Prior publications derived from the same Dryad dataset and their relationship to the present study.**

| **Study (journal)** | **Population** | **Exposure** | **N** | **Outcome** | **Follow-up** | **Main-model covariates** | **Key finding** |
| --- | --- | --- | --- | --- | --- | --- | --- |
| Masaki & Kawamoto 2019 (PLoS ONE) | Older persons with severe dysphagia requiring PEG/TPN (all etiologies) | PEG vs TPN | 253 | All-cause mortality | Enrolment Jan 2014–Jan 2017; follow-up to Feb 2018 | Propensity-matched (age, sex, comorbidities) | PEG associated with longer survival than TPN |
| Li et al. 2022 (Sci Rep) | Older patients with dysphagia (all etiologies) | Serum albumin | 253 | All-cause mortality | Same source cohort | Age, sex, and clinical covariates | Higher albumin associated with lower mortality |
| Guo et al. 2024 (Front Nutr) | Older patients with dysphagia (all etiologies) | CAR | 253 | All-cause mortality | Same source cohort | Age, sex, cerebrovascular disease, severe dementia, aspiration pneumonia, ischemic heart disease | Higher CAR associated with higher mortality |
| Pei & Wang 2025 (Front Nutr) | Older patients with dysphagia (all etiologies; stroke in 53%) | PNI | 248 | All-cause mortality | Same source cohort | Age, sex, cerebrovascular disease, severe dementia, aspiration pneumonia, IHD, PEG/TPN, oral intake recovery, hemoglobin | Lower PNI associated with higher mortality |
| Shen et al. 2025 (Front Neurol) | Older patients with dysphagia requiring nutritional support (all etiologies) | CALLY index | 253 | All-cause mortality | Same source cohort | Age, sex, neurological disease, BMI, CFS, nutrient intake, severe dementia, chronic liver disease, chronic kidney disease, total cholesterol | Lower CALLY associated with higher mortality |
| Wang & Wang 2025 (Front Neurol) | Older patients with post-stroke dysphagia (history of stroke) | CAR | 133 | All-cause mortality | Same source cohort | Age, sex, BMI, CFS, oral intake recovery, aspiration pneumonia | Higher CAR associated with higher mortality |
| **Present study (Front Neurol)** | **Older patients with post-stroke dysphagia (history of stroke)** | **PNI vs CAR (head-to-head)** | **132** | **All-cause mortality** | **Same source cohort** | **Age, sex, PEG, severe dementia, aspiration pneumonia, IHD, CFS (binary)** | **Lower PNI associated with higher mortality; PNI, but not CAR, added incremental predictive value** |

CAR, C-reactive protein/albumin ratio; PNI, prognostic nutritional index; CALLY, C-reactive protein–albumin–lymphocyte index; PEG, percutaneous endoscopic gastrostomy; TPN, total parenteral nutrition; IHD, ischemic heart disease; CFS, Clinical Frailty Scale; BMI, body mass index. All studies are analyses of the same source cohort (Dryad, doi:10.5061/dryad.gg407h1, originally published by Masaki & Kawamoto, PLoS ONE 2019), hence the identical outcome and follow-up. The present study is the first to (1) evaluate PNI specifically in a stroke-restricted (post-stroke dysphagia) cohort and (2) compare PNI and CAR head-to-head within the same cohort.

**Supplementary Table S2. Collinearity statistics (generalized variance inflation factor) for the fully adjusted model.**

| **Variable** | **GVIF** | **GVIF^(1/(2×df))** |
| --- | --- | --- |
| Age | 1.287 | 1.134 |
| Sex | 1.209 | 1.099 |
| PEG | 1.498 | 1.224 |
| Severe dementia | 1.250 | 1.118 |
| Aspiration pneumonia | 1.353 | 1.163 |
| Ischemic heart disease | 1.072 | 1.036 |
| CFS (binary) | 1.074 | 1.036 |
| PNI | 1.106 | 1.052 |

All adjusted GVIF^(1/(2×df)) values were below the threshold of 2, indicating no substantial multicollinearity among the variables in the fully adjusted model. GVIF, generalized variance inflation factor; PEG, percutaneous endoscopic gastrostomy; CFS, Clinical Frailty Scale; PNI, prognostic nutritional index.

**Supplementary Table S3. Schoenfeld residual tests of the proportional hazards assumption.**

| **Variable** | **χ²** | **df** | ***p*** |
| --- | --- | --- | --- |
| PNI | 0.69 | 1 | 0.41 |
| Age | 0.11 | 1 | 0.74 |
| Sex | 0.43 | 1 | 0.51 |
| PEG | 0.12 | 1 | 0.73 |
| Severe dementia | 0.00 | 1 | 0.99 |
| Aspiration pneumonia | 1.24 | 1 | 0.27 |
| Ischemic heart disease | 0.15 | 1 | 0.69 |
| CFS (binary) | 1.37 | 1 | 0.24 |
| Global | 4.86 | 8 | 0.77 |

(A) Fully adjusted model with PNI as a continuous variable. All *p* > 0.05, indicating the proportional hazards assumption was met.

| **Variable** | **χ²** | **df** | ***p*** |
| --- | --- | --- | --- |
| CAR | 0.58 | 1 | 0.45 |
| Age | 0.11 | 1 | 0.74 |
| Sex | 0.22 | 1 | 0.64 |
| PEG | 0.39 | 1 | 0.53 |
| Severe dementia | 0.01 | 1 | 0.93 |
| Aspiration pneumonia | 0.87 | 1 | 0.35 |
| Ischemic heart disease | 0.82 | 1 | 0.37 |
| CFS (binary) | 1.73 | 1 | 0.19 |
| Global | 6.28 | 8 | 0.62 |

(B) Fully adjusted model with CAR as a continuous variable. All *p* > 0.05, indicating the proportional hazards assumption was met. PNI, prognostic nutritional index; CAR, C-reactive protein/albumin ratio; PEG, percutaneous endoscopic gastrostomy; CFS, Clinical Frailty Scale.

**Supplementary Table S4. Sensitivity analyses for model stability: (A) Firth penalized Cox regression and (B) bootstrap internal validation.**

**(A) Firth penalized Cox regression for all-cause mortality (PNI model).**

| **Variable** | **HR** | **95% CI** | **p** |
| --- | --- | --- | --- |
| PNI (per 1-unit increase) | 0.93 | 0.90–0.97 | < 0.001 |
| Age (per 1 year) | 1.02 | 0.99–1.06 | 0.121 |
| Female sex (vs male) | 0.33 | 0.18–0.58 | < 0.001 |
| PEG (vs TPN) | 0.20 | 0.10–0.40 | < 0.001 |
| Severe dementia | 1.13 | 0.55–2.20 | 0.734 |
| Aspiration pneumonia | 1.28 | 0.66–2.43 | 0.456 |
| Ischemic heart disease | 1.92 | 1.08–3.33 | 0.027 |
| CFS ≥ 8 (vs < 8) | 4.98 | 1.57–25.06 | 0.004 |

**(B) Bootstrap internal validation (500 resamples) of the fully adjusted PNI model.**

| **Metric** | **Value** |
| --- | --- |
| Apparent C-index | 0.821 |
| Bootstrap optimism | 0.012 |
| Optimism-corrected C-index | 0.810 |

PNI, prognostic nutritional index; PEG, percutaneous endoscopic gastrostomy; TPN, total parenteral nutrition; CFS, Clinical Frailty Scale; HR, hazard ratio; CI, confidence interval. The Firth penalized model was fitted to reduce small-sample bias given 61 events and seven confounders. Bootstrap optimism is the difference between the apparent C-index and the average C-index evaluated on bootstrap samples; the small optimism (0.012) indicates the model is not materially overfit.

Supplementary Table S5. Association between PNI (alternative cut-offs [**(Scrutinio et al. 2020)**]) and mortality in different models.

| **PNI** | **Model 1 HR (95% CI), *p*** | **Model 2 HR (95% CI), *p*** | **Model 3 HR (95% CI), *p*** |
| --- | --- | --- | --- |
| < 35 (T1) | 1 (Reference) | 1 (Reference) | 1 (Reference) |
| 35–38 (T2) | 0.364 (0.150–0.885), *p* = 0.026 | 0.380 (0.154–0.936), *p* = 0.035 | 0.404 (0.148–1.107), *p* = 0.078 |
| ≥ 38 (T3) | 0.265 (0.156–0.452), *p* < 0.001 | 0.345 (0.196–0.607), *p* < 0.001 | 0.364 (0.199–0.668), *p* = 0.001 |

Model 1: Non-adjusted. Model 2: Adjusted for age, sex. Model 3: Adjusted for age, sex, percutaneous endoscopic gastrostomy, severe dementia,

aspiration pneumonia, ischemic heart disease, clinical frailty scale scores (binary). PNI: prognostic nutritional index; CI, confidence interval; HR, hazard ratio..

Supplementary Table S6. Association between PNI and mortality after excluding early deaths (within 30 days).

| **Variable** | **Model 1 HR (95% CI), *p*** | **Model 2 HR (95% CI), *p*** | **Model 3 HR (95% CI), *p*** |
| --- | --- | --- | --- |
| PNI (continuous) | 0.915 (0.882–0.949), *p* < 0.001 | 0.927 (0.888–0.966), *p* < 0.001 | 0.928 (0.889–0.969), *p* = 0.001 |
| PNI T1 (< 36.7) | 1 (Reference) | 1 (Reference) | 1 (Reference) |
| PNI T2 (36.7–42.8) | 0.749 (0.416–1.349), *p* = 0.336 | 0.726 (0.394–1.337), *p* = 0.304 | 0.523 (0.275–0.995), *p* = 0.048 |
| PNI T3 (≥ 42.8) | 0.193 (0.085–0.435), *p* < 0.001 | 0.327 (0.137–0.777), *p* = 0.011 | 0.300 (0.122–0.737), *p* = 0.009 |

Model 1: Non-adjusted. Model 2: Adjusted for age, sex. Model 3: Adjusted for age, sex, percutaneous endoscopic gastrostomy, severe dementia,

aspiration pneumonia, ischemic heart disease, clinical frailty scale scores (binary). PNI: prognostic nutritional index; CI, confidence interval; HR, hazard ratio.

Supplementary Figure S1

Assessment of the proportional hazards assumption in the fully adjusted models for PNI (A) and CAR (B) (all *P* > 0.05).

Supplementary Figure S2

Restricted cubic spline analyses of the association of PNI (A) and CAR (B) with all-cause mortality. Solid lines show hazard ratios (log scale) relative to reference values, and shaded areas represent 95% confidence intervals. The nonlinear components were not statistically significant (PNI, p = 0.99; CAR, p = 0.20), supporting the linear specification used in the primary analysis. PNI, prognostic nutritional index; CAR, C-reactive protein/albumin ratio.

Supplementary Figure S3

Calibration of the clinical model plus PNI for predicting 1-year mortality. The x-axis shows predicted 1-year mortality; the y-axis shows observed 1-year mortality estimated by bootstrap calibration (200 resamples). The dashed diagonal line indicates perfect calibration; the curve lies close to the diagonal, indicating acceptable agreement. PNI, prognostic nutritional index.

Supplementary Figure S4

Decision curve analysis for 1‑year mortality prediction using PNI and CAR. PNI: Prognostic Nutritional Index; CAR: C‑reactive protein/albumin ratio.
